# Ordinal synchronization mark sequence and its steganography for a multi-link network covert channel

**DOI:** 10.1371/journal.pone.0252813

**Published:** 2021-06-04

**Authors:** Songyin Fu, Rangding Wang, Li Dong, Diqun Yan

**Affiliations:** Faculty of Electrical Engineering and Computer Science, Ningbo University, Ningbo, China; Fuzhou University, CHINA

## Abstract

A multi-link network covert channel (MLCC) such as *Cloak* exhibits a high capacity and robustness and can achieve lossless modulation of the protocol data units. However, the mechanism of *Cloak* involving an arrangement of packets over the links (APL) is limited by its passive synchronization schemes, which results in intermittent obstructions in transmitting APL packets and anomalous link switching patterns. In this work, we propose a novel ordinal synchronization mark sequence (OSMS) for a *Cloak* framework based MLCC to ensure that the marked APL packets are orderly distinguishable. Specifically, a unidirectional function is used to generate the OSMS randomly before realizing covert modulation. Subsequently, we formulate the generation relation of the marks according to their order and embed each mark into the APL packets by using a one-way hash function such that the mark cannot be cracked during the transmission of the APL packet. Finally, we set up a retrieval function of the finite set at the covert receiver to extract the marks and determine their orders, and the APL packets are reorganized to realize covert demodulation. The results of experiments performed on real traffic indicated that the MLCC embedded with OSMS could avoid the passive synchronization schemes and exhibited superior performance in terms of reliability, throughput, and undetectability compared with the renowned *Cloak* method, especially under a malicious network interference scenario. Furthermore, our approach could effectively resist the inter-link correlation test, which are highly effective in testing the *Cloak* framework.

## Introduction

In the current cyberspace, the requirements of information security and privacy have reached unprecedented levels [1]. Generally, encryption is the key approach to prevent any unauthorized access to protected data. However, in many cases, information can be acquired without decrypting the communication content, and the unauthorized users can obtain the required information through external representation information such as the information transmission frequency [2]. In addition, the encryption form of the data may attract the monitor’s attention, who can analyze and extract various sensitive information points by monitoring the data transmission over the network and implementing malicious attacks [3]. In this context, the network covert channel (NCC) provides an alternative approach by concealing the transmission. NCC uses the normal network communication as a cover and transmits sensitive or personal information such as a key or account in insecure networks without being detected by the monitor [3, 4]. As a supplement to encryption, NCC is used in scenarios in which normal communication is extremely revealing to transmit any secret information [5].

In general, NCC utilizes the network protocol as the carrier and modulates the secret message into the header fields of the protocol data units (PDUs) or the timing of PDUs. The former and latter strategies correspond to the network covert storage channel (CSC) and covert timing channel (CTC), respectively. The two kinds of covert channels transmit the secret message along with the overt traffic and ensure that the manipulated carrier is as consistent as the original carrier as possible, in terms of the structure and flow pattern.

It is challenging for the CSC and CTC to exhibit both excellent robustness and undetectability simultaneously, and the multi-link covert channel (MLCC) has been proposed to overcome this limitation [6–9]. Instead of establishing a single link between a covert sender (CS) and covert receiver (CR), the MLCC maintains *L* links between the CS and CR, and covert codewords are mapped to the combinations of *R* packets sent over the *L* links. For example, the typical MLCC framework *Cloak* modulates a covert word to an arrangement of packets over the links (APL) and it does not involve modifying the packet head or manipulating the packet flow. Thus, the *Cloak* can achieve a high capacity by increasing *R* and *L* [9], and it can resist the conventional steganalysis approaches on each link.

However, the *Cloak* framework involves certain limitations. First, this approach utilizes the acknowledgements (ACKs) of the TCP as synchronization signals to coordinate the sending order of each TCP connection [6, 7]. Since the ACKs of the TCP are used to enhance the communication reliability, the covert communication of *Cloak* tends to disturb the overt traffic of the TCP connections [10, 11]. Second, the *Cloak* framework conveys the covert codewords by deliberately controlling the sending behaviors of the TCP connections. In this case, the connection switching pattern of the host may be altered, leading to a significant deviation from the normal concurrent scheme of the computer network (CSCN). Both of the two limitations can be attributed to the passive synchronization schemes of *Cloak*.

In this paper, we propose a novel synchronization scheme for *Cloak* framework based MLCC by introducing an ordinal synchronization mark sequence (OSMS) composed of a set of ordinal synchronization marks (OSMs) with an ordinal relationship and design a MLCC embedded with OSMS (MLCCOSMS). MLCCOSMS inherits the multi-link covert modulation scheme of *Cloak* but utilizes OSMs instead of using ACKs of TCP as synchronization signals. Thus, MLCCOSMS exhibits the same camouflage ability and capacity as those of *Cloak*. Besides, MLCCOSMS is no longer rely on the ACKs for the synchronization of covert communication while fully exploiting the reliability mechanism of the TCP for the APL packet transmission. Hence, it eliminates the impact on the overt traffic and its robustness against severe interference in a real network is considerably higher than that of *Cloak*.

Furthermore, the CS of MLCCOSMS can arbitrarily mimic the normal CSCN during the covert communication to evade the flow test and inter-link correlation test. Our approach embeds the OSM in the payload of the APL packet to pass the ANIs, owing to which, the security system on the link can protect the entire operation to a certain extent. Moreover, the OSMs are generated randomly and embedded in the APL packets irreversibly. Theoretically, an active monitor cannot crack the OSM from the APL packet and obtain any information pertaining to the secret message from the OSM, even if the OSM is exposed. The contributions of this work can be summarized as follows:

1. We clarify essential limitations of the synchronization schemes of Cloak and the risks arising from them in covert communication.
2. We introduce a requirements specification for an ideal MLCC.
3. We propose OSMS for the synchronization of covert communication of Cloak framework based MLCC, and introduce a formulation from the generation relation of OSMs in an OSMS to their orders
4. We update the synchronization schemes of Cloak by replacing ACKs with OSMs as synchronization signals and design MLCCOSMS. We also propose a secure steganography of OSMS for MLCCOSMS.

## Materials and methods

### Related works

CSC and CTC usually have low throughput due to the carrier speed limitations and the balance between the undetectability and the embedding rate of covert modulation. Furthermore, the CSC is vulnerable to active network intermediaries (ANIs) (e.g., protocol scrubbers [12] and traffic normalizers [13]). Although the concealment capacities of the CTC are more enhanced than those of the CSC [14, 15], the approach is susceptible to synchronization problems, noise and malicious attacks [16] since the CTC is built on a single unidirectional link, which makes it difficult to employ link quality feedback and reception confirmation schemes [17]. Moreover, the CTC flow uses the same link as that of the overt flow in a time-sharing manner, therefore, in principle, an active monitor can always identify the differences between the two flows to detect the existence of the CTC. In fact, the main steganalysis approaches against the CTC aim to measure the variation in the statistical characteristics of the overt traffic caused by the CTC, such approaches include the KS and KL divergence tests [18], descriptive analytics of traffic (DAT) [19], regularity and ε-similarity tests [20], entropy (EN) and corrected conditional entropy (CCE) tests [21], and auto-correlation of inter-packet delay (IPD) tests [22, 23]. Moreover, detection approaches based on deep neural networks can perform efficient counterwork [24]. Thus, it is difficult to improve the performance of the NCC built on a single unidirectional link.

**Fig 1:**
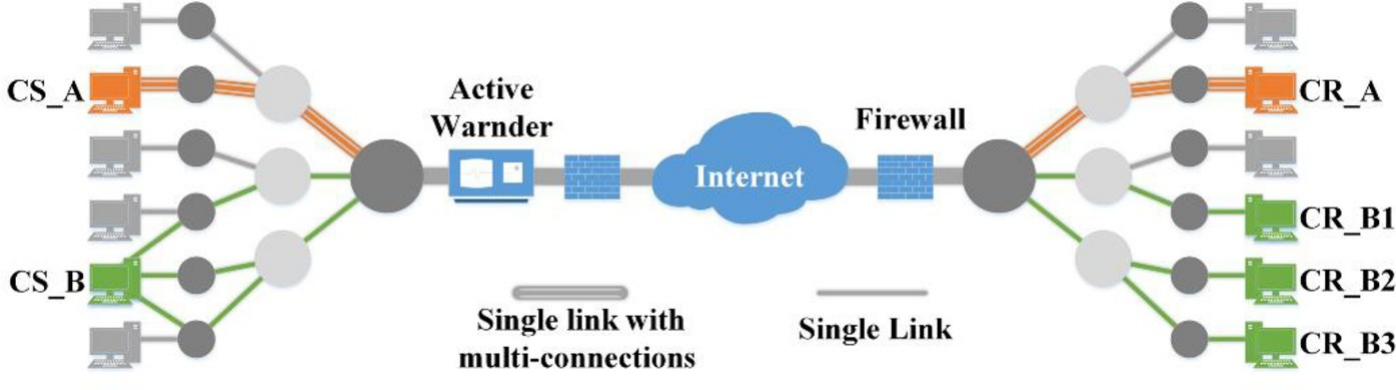
Two structures of a Multi-Link covert channel.

In contrast, the MLCC consists of multiple links. Fig 1 shows two common structures of the MLCC [7]. One structure includes a CS_A and CR_B, which maintain a physical link with multiple logic links during the covert communication. The other structure involves a distributed system with multiple CRs (CR_B and CR_B1-3), that each CR maintaining a physical link with the CS. An active monitor as well as security equipment such as network firewalls, intrusion detection systems may be deployed anywhere on the links and they can intercept, eavesdrop, and interfere with the flows at any time. Commonly, they are deployed as close as possible to the boundary of the sub-LAN being monitored [25].

Most MLCC approaches are based on the former structure owing to its simplicity and low computational cost. Khan et al. [6] used multiple active TCP connections between a pair of communicating hosts and modulated the secret codewords into the order and sequence of the connections to or from which packets were sent or received. Luo et al. [8] used the different combinations of *N* packets sent over *X* flows in each round to represent a covert codeword; subsequently, the authors optimized their approach and designed a classic MLCC known as *Cloak* [7, 9]. Zhang et al. [5] extended the idea of *Cloak* and built a covert channel by realizing packet rearrangement over mobile networks. El-Atawy et al. [26, 27] exploited the packet reordering phenomenon to make a packet sequence detectable in network flows to enhance the capacity and stealth.

The data rate provided by the *Cloak* framework is considerably higher than that of the existing CTCs as it combinatorial in nature, and its capacity monotonically increases with *R* and *L*. Luo et al. [9] explored 9 different modulation and demodulation methods for *Cloak*, based on the distinguishability and sequence detectability of the flows and packets. In these approaches, every distinct covert codeword is mapped to a unique APL. Assuming *Cloak*(*R*,*L*) has *N* different APLs, where *N* has a maximum value of *R*!(*R*−1)!*L*/(*R*−*L*)!, and its capacity is ⌊*log*_2_*N*⌋/*R* in bits per packet. The capacity may be even higher if single-link NCCs are combined with *Cloak* to establish a hybrid covert channel [28].

*Cloak* framework does not modify or manipulate the packet head or flow. Therefore, this approach can effectively evade the conventional single-link detection methods by mimicking the normal flows on each link [7] and even introducing fake links [6]. Owing to the lossless modulation, *Cloak* can be supported by many network protocols [29] and can exploit the reliability of the carriers, e.g., through a TCP-based instance, 100% reliable transmission of a secret message can be realized [7]. In the following discussion, it assumed that *Cloak* builds on TCP flows.

However, *Cloak* involves certain limitations. First, the monitor’s attention may be attracted if long-time covert communication is performed using excessively many links. Certain countermeasures scan the number of active random ports of the hosts to detect abnormal multi-link communications performed by two pairs of hosts on the network [10]. In this context, *Cloak*, which is based on HTTP [9], is inclined to be treated as malicious access if the request targets are excessively many or the request is extremely dense. Consequently, the number of links and duration of the transmission of successive APLs should be strictly constrained.

Second, *Cloak* involves the key problem of synchronization between links. An APL is represented by the number of packets on each link and their orders. When a message is being conveyed, the APL packet groups consist of intra-link and inter-link processes. Both these processes should be precisely synchronized to ensure that every APL is intact when the corresponding APL packet group is received by the CR. Liu and El-Atawy et al. [26, 30] suggested that synchronization mark (SM) can be embedded into the sequence number field of the TCP packet. Luo et al. [7] encoded the SM into window size field or the APL packet size. However, embedding SMs into the covert flows through common network steganography approaches not only consumes the partial bandwidth of *Cloak* but also weakens its reliability and undetectability, which in turn makes SMs a security bottleneck of the *Cloak* framework.

According to an alternative approach [7], the synchronization between the links of *Cloak* can be performed by following certain indications such as the TCP ACKs. However, in this scenario, the traffic of the APL packets on each single link might be different from the normal traffic. Shi et al. [10] estimated the APL group size by tracking the intermittent changes of the IPDs and counting the cycle of occurrence to detect the presence of *Cloak*. Wang et al. [11] proposed a detection method that involved testing the burst size distribution of the flows over the links. It was noted that the link switching pattern of the packet transmission over multiple links tended to be different than that of the normal CSCN when the synchronization schemes of *Cloak* were being implemented. To overcome this limitation, in this study, the root of the synchronization schemes of *Cloak* was considered.

#### Synchronization schemes of *Cloak*

According to different techniques of controlling the transmission behaviors of links [9], the synchronization schemes of *Cloak* can be classified into three types, as presented in Table 1.

**Table 1:** Classification of *Cloak* synchronization schemes.

Accuracy	Method to control the transmission behaviors of the links	Typical *Cloak*
Packet-to-Packet	The CS does not send an APL packet until it obtains the ACK from the CR for the previous packet	*Cloak*^3^
		*Cloak*^9^
Link-to-Link	The CS does not send the first packet of the *l*th link in an APL packet group until it obtains ACKs from the CR for all the APL packets of the (*l*-1)th link sent previously in the same APL packet group	*Cloak*^8^
Group-to-Group	The CS does not send the first APL packet of the *k*th APL packet group until it obtains ACKs from the CR for all the APL packets of the (*K*-1)th APL packet group sent previously	*Cloak*^5^

**Fig 2:**
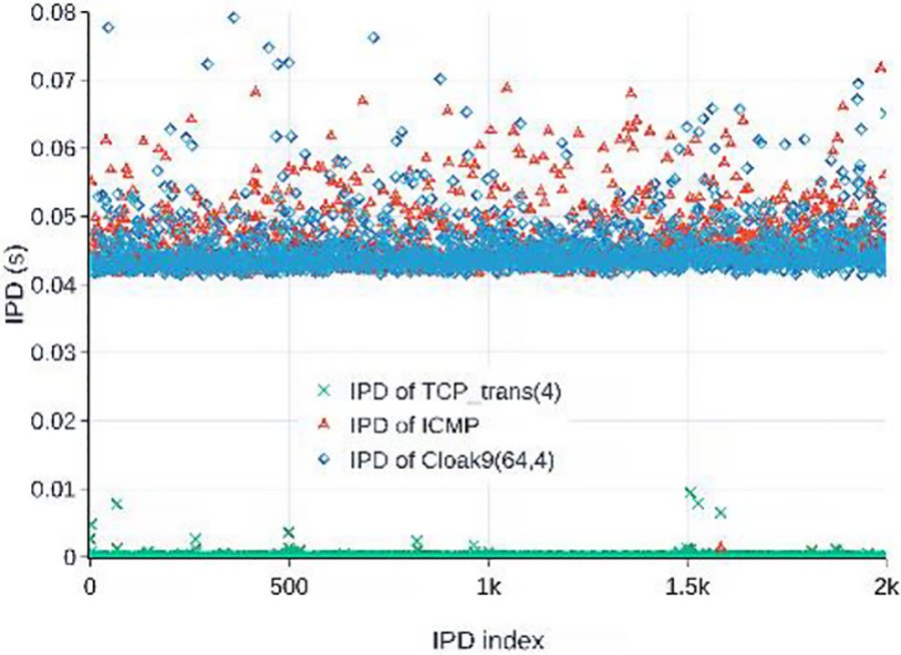
IPD samples produced by *Cloak*^9^(64,4), ping command, and TCP_trans(4) between 2 hosts. TCP_trans(4) is a channel with 4 mutually independent TCP connections between 2 hosts.

The packet-to-packet scheme has the highest capacity among the three schemes; however, it produces the biggest IPD. Assuming that the average time to transmit a packet from the CS to the CR is $\bar{T}$, which changes with the link quality, the IPD of the packet-to-packet scheme *d*_*i*_ equals $2\bar{T}+\delta_{s}+\delta_{r}$, where *δ*_*s*_ and *δ*_*r*_ denote the delay before CS and CR sending an APL packet and ACK, respectively. The time *T*_*word*_ required to transmit a covert codeword can be defined as:

$$T_{word}=\sum_{i=1}^{R}d_{i}=2\bar{T}R+\sum_{\delta_{s}=\delta_{s}^{min}}^{\delta_{s}^{max}}p_{s}(\delta_{s})\delta_{s}R+\sum_{\delta_{r}=\delta_{r}^{min}}^{\delta_{r}^{max}}p_{r}(\delta_{r})\delta_{r}R$$ (1)

where *p*_*s*_(*δ*_*s*_) and *p*_*r*_(*δ*_*r*_) are the probability density functions (PDFs) of *δ*_*s*_ and *δ*_*r*_ respectively. As shown in Fig 2, *Cloak*^9^ produces nearly the same IPDs as those of the RTT, which is considerably larger than the standard TCP flow.

The link-to-link and group-to-group schemes are compatible with the delayed ACK algorithm of the TCP [31] and demand less ACKs. Correspondingly, *T*_*word*_ can be modified as:

$$T_{word}=2\bar{T}N_{ack}+\sum_{\delta_{s}=\delta_{s}^{min}}^{\delta_{s}^{max}}p_{s}(\delta_{s})\delta_{s}R+\sum_{\delta_{r}=\delta_{r}^{min}}^{\delta_{r}^{max}}p_{r}(\delta_{r})\delta_{r}N_{ack}$$ (2)

where *N*_*ack*_ is the number of ACKs sent by the CR during one APL packet group transmission. *T*_*word*_ increases with *N*_*ack*_ and (2) transforms to (1) when *N*_*ack*_ equals *R*. Moreover, a smaller *N*_*ack*_ leads to a higher goodput of *Cloak*.

#### Risks pertaining to the synchronization schemes

**Fig 3:**
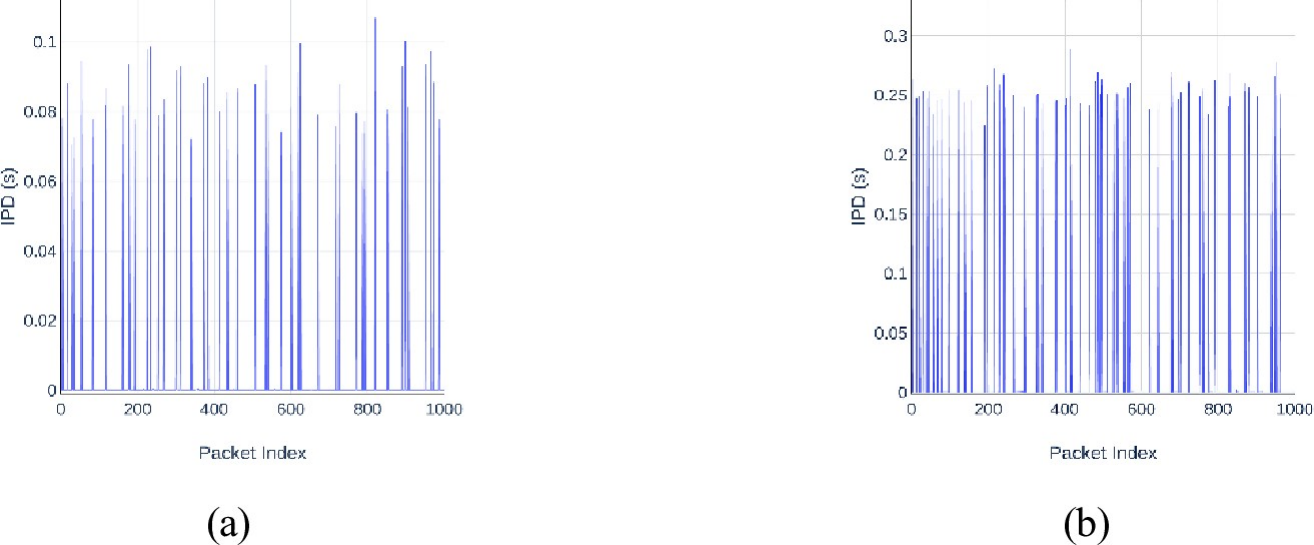
IPDs of *Cloak* link. **(a) *Cloak***^**5**^**(64,4). (b) *Cloak***^**8**^**(64,4)**.

Sending ACKs repeatedly from the CR to the CS is inefficient and can potentially attract a monitor’s suspicion. Thus, the packet-to-packet scheme is not practical. Furthermore, because the CS cannot exactly predict the correct time to send the next APL packet due to the unpredictable *p*_*r*_(*δ*_*r*_) and the unstable link quality, it passively waits for the ACK of the previous APL packets. Consequently, the discontinuity of the APL packet flows between the APL packet groups inevitably occurs in the link-to-link and group-to-group schemes. The intermittent IPD alteration of the APL packets is shown in Fig 3.

**Fig 4:**
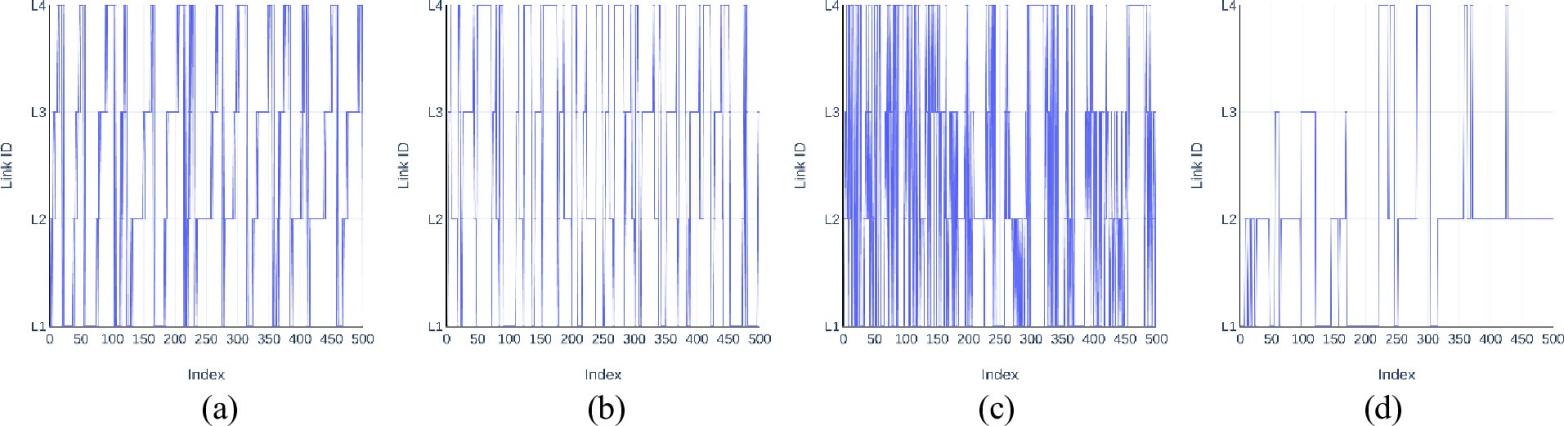
Link switching time series of *Cloak*^9,8,5^(32,4) and TCP_trans(4). **(a) *Cloak***^**9**^**(32,4)**. (b) ***Cloak***^**8**^**(32,4)**. **(c) *Cloak***^**5**^**(32,4)**. (d) TCP_trans(4).

Furthermore, the CS tends to generate a link switching pattern which is different from that of the normal CSCN, due to the nature of the covert modulation. Fig 4 shows that the link switching time series of four mutually independent TCP connections between two hosts is relatively simple when all the four connections perform the transmission simultaneously under a normal CSCN. However, the time series of the three kinds of *Cloak* involve significant fluctuations.

#### Challenges

All the problems of *Cloak* can be attributed to the multiplexing of the ACKs, that *Cloak* use ACKs as indicators for both the link quality and synchronization between links, whereas the two goals have different execution rhythms. We consider that an ideal MLCC must satisfy the following requirements:

1. Use the reliability mechanism of the carrier channel as much as possible to ensure reliability.
2. Robust to both stochastic and malicious interferences, with a certain fault tolerance, and the demodulation failure of a few codewords should not affect the others.
3. Restraint in the number of links and covert transmission time to enhance the concealment.
4. Try to make the covert modulation and demodulation as lossless as possible to the carrier and can resist active attacks on the channel. Specifically, the attacker should not be able to crack any hidden information, even if the APL packet is intercepted.
5. There is no effect on the concurrent behaviors of the host.

### MLCCOSMS

#### Preliminary knowledge

Before describing the specific method, we present the meanings of certain symbols (see Table 2) and two definitions.

**Table 2:** Notations.

Symbols	Meaning
A	set of distinct APLs
W	set of distinct codewords
ℤ(*z*)	set of positive integers from 1 to z
$\tilde{M}$	successive ordinal marker sequence with length *R*
$\bar{M}$	set of ordinal marks
${\bar{\bar{M}}}^{neg}$	set of type marks of a negotiation
${\bar{\bar{M}}}^{seq}$	set of type marks of an ordinal
*w*_*j*_	covert codeword with sequence number *j* in the secret message
${\bar{M}}_{j}^{w}$	set of ordinal marks for the APL packet group corresponding to codeword *w*_*j*_
${\bar{M}}_{j}^{c}$	set of ordinal marks for the APL packets belonging to the APL packet group corresponding to *w*_*j*_
*A*_*w*_	APL corresponding to codeword *w*
*AML*_*w*_	arrangement of marks over the links (AML) corresponding to *A*_*w*_
${\bar{\bar{key}}}_{rand}^{neg}$	random type mark of the negotiation in ${\bar{\bar{M}}}^{neg}$
${\bar{\bar{key}}}_{rand}^{seq}$	random type mark of the ordinal in ${\bar{\bar{M}}}^{seq}$
${\bar{key}}_{rand\_w\_j}^{seq}$	random ordinal mark in ${\bar{M}}_{j}^{w}$
${\bar{key}}_{i}^{seq}$	ordinal mark with sequence number *i* in ${\bar{M}}_{j}^{c}$
*F*(*x*)	mapping function from the codewords to the APLs, *A*_*w*_ = *F*(*w*), $A_{w}\in A$, w∈W

*Definition 1 (generation relation of marks)*. Given the marks *key* and *key*’, if there exists an one-way function *g*(*x*), *key*’ can be obtained from *key* by performing a finite number of iterations on *g*(*x*); in other words, *key*’ = *g*(…(*g*(*g*(*key*)))). Consequently, *key* and *key*’ exhibit a generation relation with *g*(*x*), wherein the *key* is the original mark and *key*’ is the derived mark and formalized as *key*’ = *g*’(*key*), where *t* is the number of iterations. In addition, *key* is regarded as the seed of *key*’ when *t* = 1.

*Definition 2* (*ordering relation of marks*). Suppose *key* and *key*’ satisfy the generation relation of *g*(*x*), where *key* and *key*’ denote the original and derived mark, respectively. Subsequently, they satisfy the ordering relation as *key* before *key*’, formalized as (key,key')→.

#### OSMS

According to *Definition 1*, given a seed *key*_0_, a sequence of marks $\bar{M}$ sized *K* can be generated by performing *K* iterations of *g*(*x*) for *key*_0_. Here $\bar{M}=\{key_{1},\cdots ,key_{i},\cdots ,key_{j},\cdots |i,j\in [1,k]\}$, and the generation relation between the marks in $\bar{M}$ can be defined as:

$$key_{j}=\{\begin{array}{c} g(key_{0})\;i=1\\ g^{j-i}(key_{i})2\leq i<j\leq K\\ key_{i}\;only\;i=j \end{array}$$ (3)

Thus, every mark in $\bar{M}$ is distinct, and the marks in $\bar{M}$ can be formulated as an ordinal mark sequence with an arbitrary length since every two marks *key*_*i*_ and *key*_*j*_ satisfy $\vec{\left({\mathrm{key}}_{i},{\mathrm{key}}_{j}\right)}$ if *i* < *j*, according to *definition 2*.

#### Covert modulation and demodulation

**Fig 5:**
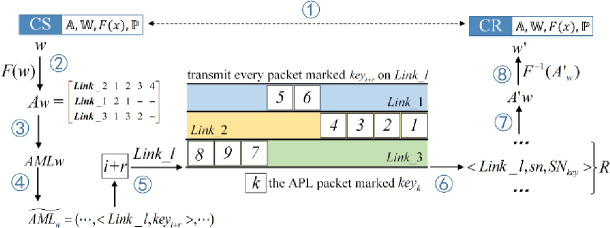
Process of covert codeword transmission of the MLCCOSMS.

The MLCCOSMS utilizes an ordinal mark sequence derived from $\bar{M}$ as the synchronization mark. Fig 5 illustrates the eight-step process of the covert codeword transmission of the MLCCOSMS.

The CS shares a group of parameter sets with the CR secretly in advance. Before sending the covert codewords, the CS generates $\bar{M}$ for the entire secret message through ℙ and covertly directs the CR to generate a copy of $\bar{M}$ after all the *L* links have been established. During the covert transmission, the CS converts each codeword to a group of APL packets then marks them with corresponding OSMs and sends them to the CR on specific links one by one. CR reorganizes the packets according to their arrival orders, receiving links and the ordering relation of embedded marks, and rebuilds the APL of each group of packets, then demodulates the codeword.

#### OSMS generation

The information of the positions of an APL packet in one APL packet group and among different APL packet groups should be indicated in the marks. In addition, the marks need to be updated to improve the channel security. In this context, the MLCCOSMS extends the marks to type and ordinal marks. The type marks are classified as type marks of the negotiation and ordinal. The ordinal and negotiation type marks are used to sort the APL packets and generate the ordinal marks, respectively. All kinds of marks are binary sequences with a fixed length.

The CS and CR share a parameter set $\mathbb{P}=\{{\bar{\bar{M}}}^{neg},{\bar{\bar{M}}}^{seq},\mathbb{Z}(z),g(x),H(x),N_{w},I\}$, when MLCCOSMS is established, *N*_*w*_ is the size of the secret message block in the codeword, *I* is a redundant parameter (*I* ≥ 2*R*), and all the elements in ${\bar{\bar{M}}}^{neg}$ and ${\bar{\bar{M}}}^{seq}$ are different. Before sending a new block of secret messages, the CS selects a ${\bar{\bar{key}}}_{rand}^{neg}$ in ${\bar{\bar{M}}}^{neg}$ randomly as the seed to generate $\bar{M}$, as described in (4), where ${\bar{key}}_{1,1}^{seq}=g({\bar{\bar{key}}}_{rand}^{neg})$, ${\bar{key}}_{j,i}^{seq}=g({\bar{key}}_{j,i-1}^{seq})$, ${\bar{key}}_{j,1}^{seq}=g({\bar{key}}_{j-1,I}^{seq})$, $\vec{{\bar{\mathrm{key}}}_{j,i‐1}^{\mathrm{seq}},{\bar{\mathrm{key}}}_{j,i}^{\mathrm{seq}}}$, $\vec{{\bar{\mathrm{key}}}_{j‐1,i}^{\mathrm{seq}},{\bar{\mathrm{key}}}_{j,1}^{\mathrm{seq}}}$, and $\vec{{\bar{\mathrm{key}}}_{j‐1,I}^{\mathrm{seq}},{\bar{\mathrm{key}}}_{j,1}^{\mathrm{seq}}}$ are satisfied.


$$\bar{M}=[\begin{array}{ccc} {\bar{key}}_{1,1}^{seq} & \cdots & {\bar{key}}_{1,I}^{seq}\\ \vdots & \ddots & \vdots \\ {\bar{key}}_{N_{w},1}^{seq} & \cdots & {\bar{key}}_{N_{w},I}^{seq} \end{array}]$$ (4)

The CS selects a random positive integer *m* in [*R*, *I-R*] from ℤ(*z*) and separates each row set of $\bar{M}$ into two ordinal mark sub-sets, ${\bar{M}}_{j}^{w}=\{{\bar{key}}_{j,i}^{seq}|i=1,\cdots ,m\}$, ${\bar{M}}_{j}^{c}=\{{\bar{key}}_{j,i}^{seq}|i=m+1,\cdots ,I\}$. Both correspond to the APL packet group index, and APL packet indexes in the group respectively, *j* is the row index of $\bar{M}$.

#### OSMS embedding and extraction

The CS first covertly directs the CR to generate a copy of $\bar{M}$ by sending a packet embedded with negotiation type marks *P*^*neg*^(*Data*^*neg*^) to CR on an arbitrary link, where *Data*^*neg*^ is the packet payload, *Data*^*neg*^ = *Data*‖*MT_Mac*‖*MW_Mac*‖*MC_Mac* and $MT\_Mac=H(Data\|{\bar{\bar{key}}}_{rand}^{neg})$, $MW\_Mac=H({\bar{\bar{key}}}_{rand}^{neg}\|m)$, $MC\_Mac=H({\bar{\bar{key}}}_{rand}^{neg}\|r)$, (*r*∈ℤ(*z*),*r*≠*m*). The CR extracts ${\bar{\bar{key}}}_{rand}^{neg'}$ and *m*’ from the payload of the received packet by implementing ${\bar{\bar{key}}}_{rand}^{neg'}=Exit(Data',{\bar{\bar{M}}}^{neg},MT\_Mac')$ and $m'=Exit({\bar{\bar{key}}}_{rand}^{neg'},\mathbb{Z}(z),MW\_Mac')$ respectively. In theory, ${\bar{\bar{key}}}_{rand}^{neg'}={\bar{\bar{key}}}_{rand}^{neg}$, and *m*’ = *m* if no error occurs during the transmission of *P*^*neg*^(*Data*^*neg*^). Therefore, the copies of $\bar{M}$, ${\bar{M}}_{j}^{w}$, and ${\bar{M}}_{j}^{c}$ denoted as M¯', M¯jw', M¯jc', respectively, can be produced.

During the covert codewords transmission, the CS fetches the corresponding ${\bar{M}}_{j}^{w}$ and ${\bar{M}}_{j}^{c}$ for every codeword *w*_*j*_ of the secret message block sequentially, and embedded the payload of each APL packet *Data*^*seq*^ with type mark of the ordinal and ordinal mark, where $MT\_Mac=H(Data\|{\bar{\bar{key}}}_{rand}^{seq})$, $MW\_Mac=H(Data\|{\bar{key}}_{rand\_w\_j}^{seq})$, and $MC\_Mac=H(Data\|{\bar{key}}_{i+r}^{seq})$, It must be noted that the choice of ${\bar{\bar{key}}}_{rand}^{seq}$ and ${\bar{key}}_{rand\_w\_j}^{seq}$ is unique for every APL packet in an APL packet group. In step ⑥, the CR implements ${\bar{\bar{key}}}_{rand}^{seq'}=Exit(Data',{\bar{\bar{M}}}^{seq},MT\_Mac')$ to extract ${\bar{\bar{key}}}_{rand}^{seq'}$, which indicates that the received packet is an APL packet with the ordinal marks. In this manner, the embedded ordinal mark ${\bar{key}}_{rand\_w\_j}^{seq'}$ for the APL packet group can be obtained after employing $Exit(Data',{\bar{M}}_{j}^{w'},MW\_Mac')$. Next, ${\bar{key}}_{i+r}^{seq'}=Exit(Data',{\bar{M}}_{j}^{c'},MC\_Mac')$ can be rapidly calculated since *j* is a definite value. The embedding and extraction of the OSMS is shown in algorithms 1 and 2.

**Algorithm 1** Embedding of the OSMS at the CS

**for** each secret message block

    pick ${\bar{\bar{key}}}_{rand}^{neg}$ randomly from ${\bar{\bar{M}}}^{neg}$, pick *m* and *r* randomly from ℤ(*z*) and generate $\bar{M}$

    select a link randomly and produce *P*^*neg*^(*Data*)

    $MT\_Mac\leftarrow H(Data\|{\bar{\bar{key}}}_{rand}^{neg})$, $MW\_Mac\leftarrow H({\bar{\bar{key}}}_{rand}^{neg}\|m)$, $MC\_Mac\leftarrow H({\bar{\bar{key}}}_{rand}^{neg}\|r)$

       send *P*^*neg*^(*Data*‖*MT*_*Mac*‖*MW*_*Mac*‖*MC*_*Mac*)

    **for** each covert codeword *w*_*j*_ in the covert message block

        obtain ${\bar{M}}_{j}^{w}$ and ${\bar{M}}_{j}^{c}$ and produce the corresponding APL packet group

        **for** each APL packet *P*^*seq*^(*Data*) in the group

            pick ${\bar{\bar{key}}}_{rand}^{seq}$ and ${\bar{key}}_{rand\_w\_j}^{seq}$ randomly from ${\bar{\bar{M}}}^{seq}$ and ${\bar{M}}_{j}^{w}$, respectively, and obtain ${\bar{key}}_{i+r}^{seq}$ from ${\bar{M}}_{j}^{c}$

            $MT\_Mac\leftarrow H(Data\|{\bar{\bar{key}}}_{rand}^{seq})$, $MW\_Mac\leftarrow H(Data\|{\bar{key}}_{rand\_w\_j}^{seq})$, $MC\_Mac\leftarrow H(Data\|{\bar{key}}_{i+r}^{seq})$

             send *P*^*seq*^(*Data*‖*MT*_*Mac*‖*MW*_*Mac*‖*MC*_*Mac*)

        **end for**

    **end for**

**end for**

**Algorithm 2** Extraction of the OSMS at the CR

**for** each link

    **if** a packet is received **then** obtain *Data’*, *MT_Mac’*, *MW_Mac’*, and *MC_Mac’* from the payload

        ${\bar{\bar{key}}}_{rand}^{neg'}\leftarrow$ *Exist*(*Data*’, ${\bar{\bar{M}}}^{neg}$, *MT*_*Mac*’)

        **if** ${\bar{\bar{key}}}_{rand}^{neg'}==NA$ **then** ${\bar{\bar{key}}}_{rand}^{seq'}\leftarrow$ *Exist*(*Data*’, ${\bar{\bar{M}}}^{seq}$, *MT*_*Mac*’)

            **if** ${\bar{\bar{key}}}_{rand}^{seq'}!==NA$

    **then for** *j* = 1 **to** a row count of M¯'

                ${\bar{key}}_{rand\_w\_j}^{seq'}\leftarrow$ *Exist*(*Data*’, ${\bar{M}}_{j}^{w'}$, *MW*_*Mac*’)

            **if** ${\bar{key}}_{rand\_w\_j}^{seq'}!=NA$ **then** ${\bar{key}}_{i+r}^{seq'}\leftarrow$ *Exist*(*Data*’, ${\bar{M}}_{j}^{c'}$, *MC*_*Mac*’)

                **end for**

        **else** *m*’**←***Exist* (${\bar{\bar{key}}}_{rand}^{neg'}$, ℤ(*z*), *MW*_*Mac*’)

            **if** *m*’! = *NA* then **generate** M¯'

**end for**

#### Function *Exist*(*D*, *M*, *mac*)

The main process of *Exist*(*D*,**M**,*mac*) iterates through all the marks in **M** to determine a mark *key* that satisfies *mac* = *H*(*D*||*key*). Both ${\bar{\bar{M}}}^{neg}$ and ${\bar{\bar{M}}}^{seq}$ are two finite sets assigned before the MLCCOSMS is established between the CS and CR. No pair of elements in or between the two sets is the same or has a generation relation. Thus, the elements in $\bar{M}$ are different from those of ${\bar{\bar{M}}}^{neg}$ and ${\bar{\bar{M}}}^{seq}$. As $\bar{M}$ is generated from ${\bar{\bar{M}}}^{neg}$ and has a fixed size, $Exist(Data,\bar{M},Mac)$ becomes a convergence function and can be used to obtain a clear result regarding whether $\bar{M}$ contains an unique eligible mark, in polynomial time. The CR uses this function to extract the mark embedded in an APL packet. The parameter *D* is a binary data that corresponds to the original payload of the packet when it is produced.

## Results and discussion

### Experimental results and performance evaluation

#### Reliability

MLCCOSMS fully inherits the reliability advantages of the *Cloak* regarding the APL packet transmission on every single link. Therefore, this framework satisfies requirement 1) defined in the section of challenges. Simultaneously, each APL packet is embedded with two ordinal synchronization marks that belong to an OSMS generated successively. Thus, all the APL packets can be reorganized even if some of them are out of order over the links. If certain APL packets are lost, errors only appear in the demodulation of the related APL packet groups to the corresponding codewords, not spread to other codewords. Moreover, the delay of the packets does not affect the subsequent demodulation if the CR simply establishes enough buffers. In this manner, the MLCCOSMS supports staggered demodulation and breakpoint retransmission of the APL packets. Therefore, the MLCCOSMS satisfies requirement 2) defined in the section of challenges in theory, and this aspect was tested in an experiment.

We implemented a TCP based MLCCOSMS(32,4) between two hosts. The CS host is deployed in our campus network in the Ningbo university located in the east of China. The CR host is a cloud server deployed in Guangzhou City in the south of China. In each round, we delivered 200 secret messages through the covert channel, each of which consisted of 500 random codewords. We computed the transmission success rate (TSR) of the secret messages under various kinds of interferences with different levels, all the interferences were randomly added over the links of the channel. As the continuous packet retransmissions caused by the transmission failure of the APL packets may increase the risk of detection, the transmission timeout was set as 1 minute, and a maximum of 5 reconnections were allowed in each round.

Table 3 shows that the TSR is 100% when the covert channel confronts all the levels of delay and out of order interferences as well as normal levels of packet loss. In addition, the TSR decreases gradually as the packet loss rate increases by more than 25%, even so, TSR is reasonable high when the packet loss rate increases to 50%.

**Table 3:** TSR of MLCCOSMS(32,4) under three kinds of interferences with different levels.

Packet delay		Packet out of order		Packet loss	
Delay(%RTTavg)	TSR(%)	Rate(%)	TSR(%)	Rate(%)	TSR(%)
no delay	100	0	100	0	100
0–25	100	5	100	5	100
25–50	100	10	100	10	100
50–75	100	15	100	15	100
75–100	100	20	100	20	100
100–125	100	25	100	25	99.5
125–150	100	30	100	30	99.5
150–175	100	35	100	35	98
175–200	100	40	100	40	95.2
200–225	100	50	100	50	88.3
Round-Trip Time (RTT) RTT_min_ = 32ms, RTT_max_ = 356ms, RTT_avg_ = 35.73ms, RTT_stddev_ = 7.17ms					

**Fig 6:**
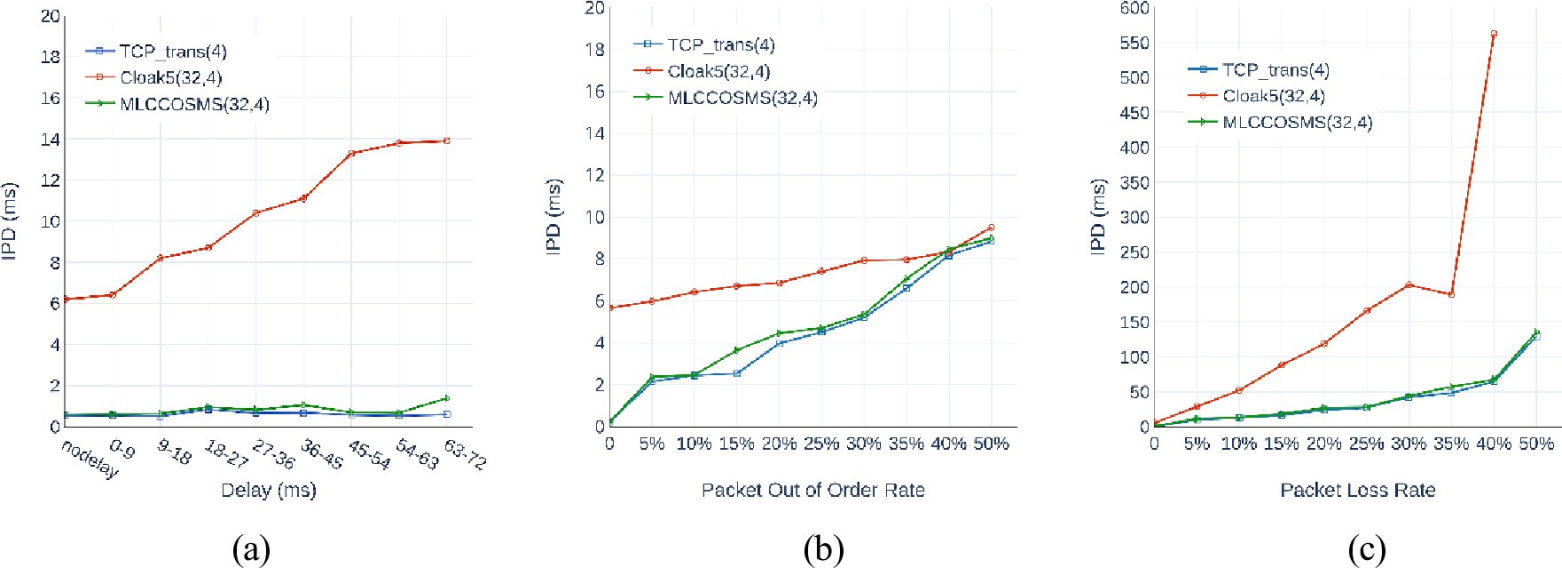
Comparison of the influence of different interferences with different levels on the IPDs of MLCCOSMS(32,4), *Cloak*^5^(32,4), and TCP_trans(4). (a) interference by delays. (b) interference by out of order mechanisms. (c) interference by packet loss.

Therefore, the MLCCOSMS operates effectively in the actual network. Furthermore, we compared the IPDs of MLCCOSMS(32,4) with those of *Cloak*^5^(32,4) and TCP_trans(4) implemented between the same two hosts. Fig 6 shows that all the levels of delay interferences only slightly influence the IPDs of MLCCOSMS (32,4). Although the IPDs gradually increase with the increase in the out of order and packet loss interferences level, the curves basically match those of TCP_trans(4). Thus, the MLCCOSMS exhibits a strong camouflage capability in terms of the IPDs. Relatively, the IPDs of *Cloak*^5^(32,4) are larger than those of MLCCOSMS (32,4) and TCP_trans(4) under various situations, and sensitive to the interferences.

#### Throughput

According to Eqs (1) and (2), the throughput of *Cloak* based on the packet-to-packet synchronization scheme and is: $TH_{Cloak}^{fine\_ack}=\frac{C_{Cloak}}{2\bar{T}+\sum_{\delta_{s}=\delta_{s}^{min}}^{\delta_{s}^{max}}p_{s}(\delta_{s})\delta_{s}+\sum_{\delta_{r}=\delta_{r}^{min}}^{\delta_{r}^{max}}p_{r}(\delta_{r})\delta_{r}}$ (bits/s) and that of the link-to-link and group-to-group synchronization schemes is: $TH_{Cloak}^{delay\_ack}=\frac{C_{Cloak}}{2\bar{T}N_{ack}/R+\sum_{\delta_{s}=\delta_{s}^{min}}^{\delta_{s}^{max}}p_{s}(\delta_{s})\delta_{s}+(\sum_{\delta_{r}=\delta_{r}^{min}}^{\delta_{r}^{max}}p_{r}(\delta_{r})\delta_{r}N_{ack})/R}$ (bits/s) where *C*_*Cloak*_ is the capacity of *Cloak*, *N*_*ack*_≥1. The covert transmission of MLCCOSMS is unidirectional due to its independence on the ACKs. So, the throughput of the MLCCOSMS can be defined as: $TH_{MLCCOSMS}=\frac{C_{Cloak}}{\bar{T}/R+\sum_{\delta_{s}=\delta_{s}^{min}}^{\delta_{s}^{max}}p_{s}(\delta_{s})\delta_{s}}$ (bits/s). It is clear that $TH_{MLCCOSMS}>TH_{Cloak}^{delay\_ack}>TH_{Cloak}^{fine\_ack}$.

Table 4 shows that the *T*_*word*_ of MLCCOSMS(64, *L*) is less than that of the other three *Cloaks* with the same value of *L*. Since the MLCCOSMS is built on only one IP link, the *T*_*word*_ of MLCCOSMS(64, *L*) is rarely affected by the increase in the number of links. Thus, the MLCCOSMS satisfies the requirement 3) specified in the section of challenges while achieving a higher and more stable throughput than that of *Cloak*. The marks embedded in the APL packet is also a special type of covert information. Therefore, the capacity of MLCCSMS per packet is even higher.

**Table 4:** Durations of conveying a codeword by several *Cloak*s and MLCCOSMS with different numbers of links.

*L*	*Cloak*^9^(64,*L*)	*Cloak*^8^(64,*L*)	*Cloak*^5^(64,*L*)	MLCCOSMS(64,L)
2	2593.22	150.92	54.29	14.37
3	2602.78	225.17	55.28	15.62
4	2627.11	296.75	55.95	15.98
5	2652.07	357.88	58.95	16.83
6	2650.48	463.42	60.64	16.58
Unit: ms				

#### Undetectability

*APL packet and mark security analysis*. The MLCCOSMS must resist malicious attacks such as interception, steganalysis, forgery, and replay in addition to guaranteeing the channel robustness in an open network. To satisfy the requirement 4) described in the section of challenges, the MLCCOSMS must satisfy 4 criteria.

S1. The CS must eliminate the interference in the modulation and mark embedding on the structure and flow of the APL packets.
S2. The CS ensure the safety of the marks and ensure that they cannot be extracted or cracked by any third party.
S3. The CR must ensure that the received APL packets arrive from the real CS by identifying the source of the received packets.
S4. The CR must ensure that the APL packets have not been tampered with or forged by analyzing the integrity of the received packets.

The non-destructive nature of the MLCC modulation method on a single link has been analyzed and demonstrated in depth in [6, 7], therefore, the proof of S1 is not presented in this work. In the context of S2, the security of the location in which the mark is embedded in the APL packet has been discussed in the section of OSMS Embedding and Extraction. Also, the validity of the main three stages of the MLCCOSMS considering S2–S4 can be proved by using the classical GNY reasoning method. (please refer to the S1 Appendix for the proofs and derivations)

*Evading the detection of the inter-link correlation test*. Since the MLCCOSMS is insensitive to packet delays, the APL packets can be sent freely without the restriction of their order in the sequenced APL packet groups, and this aspect does not significantly influence the network concurrent scheduling mechanism of the host. Therefore, the MLCCOSMS satisfies requirement 5) described in the section of challenges in theory.

**Fig 7:**
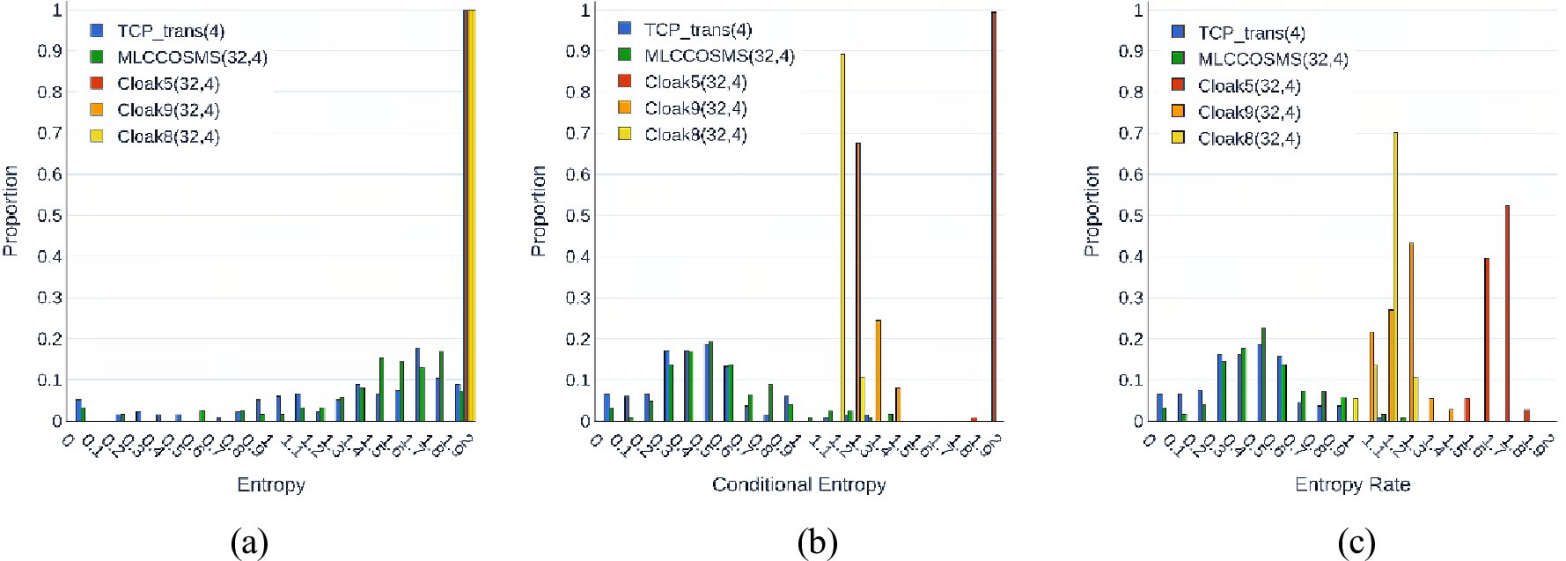
Histogram of different entropies of the five kinds of TSLS segments (*W*_*p*_ = 1000). (a) **entropy histogram of *λ***_***ch***_**.** (b) **conditional entropy histogram of *λ***_***ch***_**.** (c) **entropy rate of *λ***_***ch***_.

We implemented TCP_trans(4), MLCCOSMS(32,4), *Cloak*^9^(32,4), *Cloak*^8^(32,4), and *Cloak*^5^(32,4) on the two hosts, and recorded the time series of the link switching (TSLS) at the CS host during the process of the five channels transmitting 10K random covert codewords. Each TSLS was denoted as $\lambda_{ch}=\{L_{ch}^{1},\cdots ,L_{ch}^{i},\cdots \}$, where $L_{ch}^{i}$ is the identifier of the link on which the *i*th packet was sent, and *ch* is the identifier of the channel. We split *λ*_*ch*_ into segments, each of which contained *W*_*p*_ consecutive samples. The histogram of the entropies of the segments belonging to the five TSLSs is shown in Fig 7A, where *W*_*p*_ is set as 1000. The segment entropies of all the three *Cloak*s were concentrated in (1.9,2), which indicated that each of the four links had an equal opportunity to send an APL packet. This aspect is associated with the use of random covert codewords, and the correlation of the packet transmission behaviours between the links is affected by the correlation of the covert codewords due to the passive synchronization schemes of *Cloak*. It was noted that the entropy of the *λ*_*MLCCOSMS*(32,4)_ and *λ*_*TCP_trans*(4)_ segments is not only smaller but also more distributed, which means that MLCCOSMS can eliminate the influence of the correlation of the covert codewords. The histogram of the conditional entropy shown in Fig 7B clearly illustrates this point. Furthermore, we adopted the entropy rates [21] of the five *λ*_*ch*_, denoted as $\bar{H}(\lambda_{ch})$, to evaluate the link selection continuity of the five channels.

$$\bar{H}(\lambda_{ch})=min[CE(L_{ch}^{n}|L_{ch}^{1},\cdots ,L_{ch}^{n-1})+perc(L_{ch}^{n})\cdot EN(L_{ch}^{1})],n>1$$ (5)

where $CE(L_{ch}^{n}|L_{ch}^{1},\cdots ,L_{ch}^{n-1})$ is the *n* order condition entropy of a *λ*_*ch*_ segment. We set *n* = 20 in this paper. $perc(L_{ch}^{n})$ is the percentage of unique patterns of length *n* in a *λ*_*ch*_ segment, and $EN(L_{ch}^{1})$ is entropy of the segment. Fig 7C shows that the distribution of $\bar{H}(\lambda_{MLCCOSMS(32,4)})$ does not overlap with that of $\bar{H}(\lambda_{Cloak^{5,8,9}(32,4)})$, *Cloak*^5,8,9^ is the abbreviation of *Cloak*^5^, *Cloak*^8^ and *Cloak*^9^. However, all the distributions of the entropy, conditional entropy and entropy rate of the *λ*_*MLCCOSMS*(32,4)_ segments are similar to those of the *λ*_*TCP_trans*(4)_ segments, so, it is difficult for the monitor to identify the MLCCOSMS from the common multi-link transmission between two hosts by testing the inter-link correlation of the TSLS in a short term.

**Fig 8:**
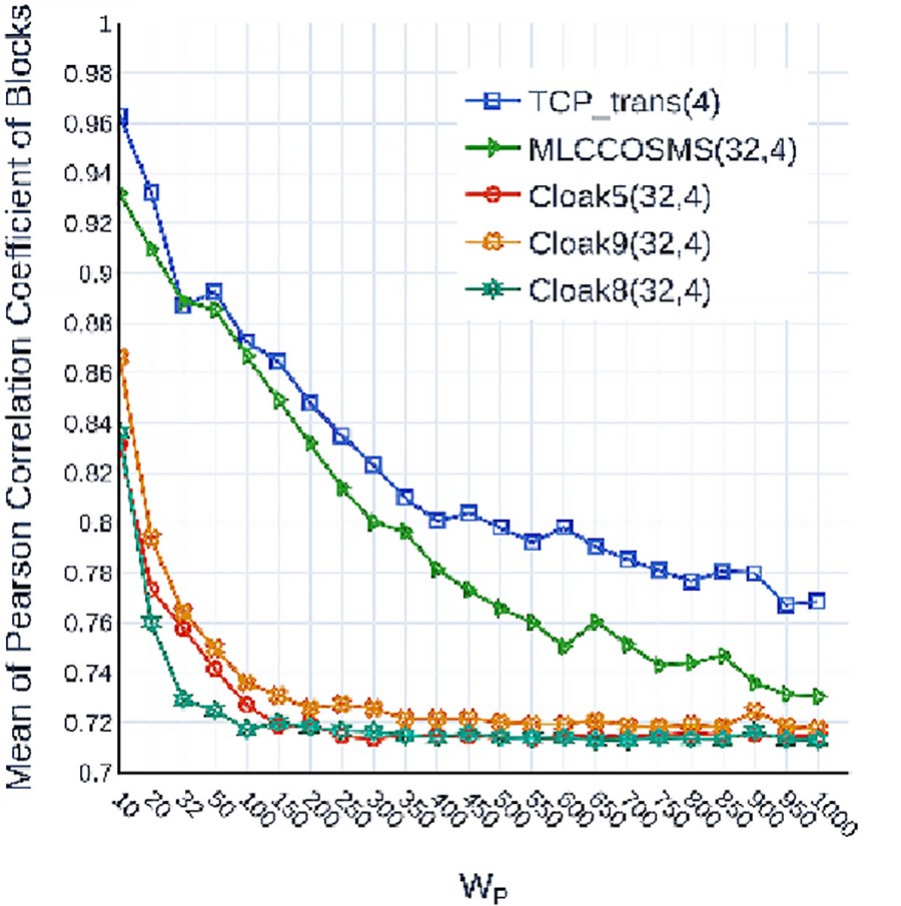
Mean of the *Pr*_*ch*_ of the five kinds of TSLSs with different *W*_*p*_.

Moreover, we investigated the interferences of the aforementioned four covert channels to the concurrent scheduling mechanism of the host in the long term by considering the Pearson correlation coefficients (PCC) of the segments. We defined the PCC set of *λ*_*ch*_ as *Pr*_*ch*_, with $Pr_{ch}=\{\mathrm{Pr}(\lambda_{ch}^{wi},\lambda_{ch}^{wj},W_{p})\}$, where Pr() returns the PCC value of two segments, *w*_*i*_ and *w*_*j*_ denote the indexes of the two segments in *λ*_*ch*_. Fig 8 shows the means of the five *Pr*_*ch*_ with different *W*_*p*_. The means of the three $Pr_{Cloak^{5,8,9}(32,4)}$ decrease considerably as *W*_*p*_ increases, due to the presence of the random covert codewords, then they are stabilized by the finite number of the covert codewords when *W*_*p*_ is large. On the contrary, the evolution of *Pr*_*MLCCOSMS*(32,4)_ is considerably more similar to that of *Pr*_*TCP_trans*(4)_.

**Fig 9:**
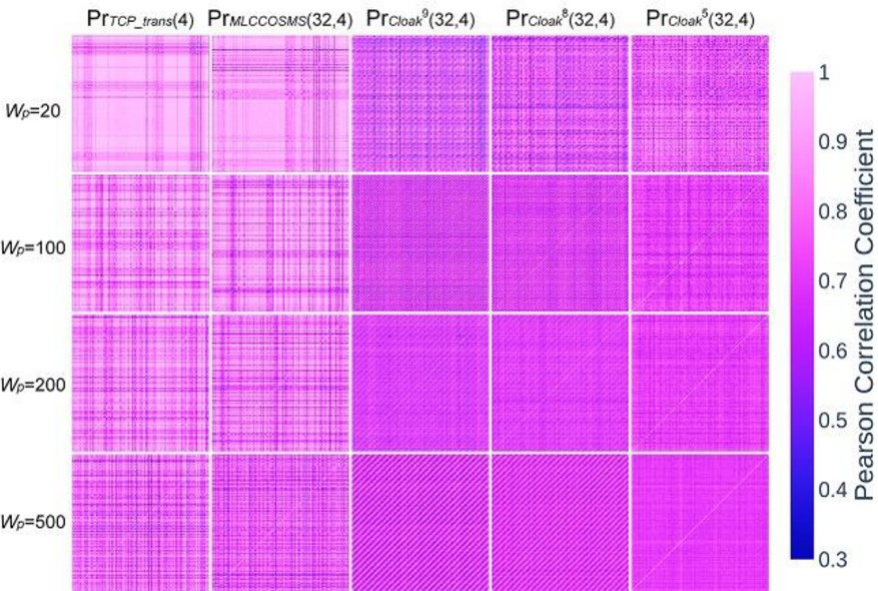
Heatmaps of *Pr*_*TCP_trans*(4)_, *Pr*_*MLCCOSMS*(32,4)_, and $Pr_{Cloak^{5,8,9}(32,4)}$ for segments of various sizes.

Finally, we plotted the five *Pr*_*ch*_ matrixes of 200 consecutive *λ*_*ch*_ segments with 4 different sizes of *W*_*p*_ by using heatmaps, as shown in Fig 9. Owing to the influence of the randomness of the covert codewords, all the three $Pr_{Cloak^{5,8,9}(32,4)}$ were lower than *Pr*_*TCP_trans*(4)_ and exhibited different patten of value change from *Pr*_*TCP_trans*(4)_, while The similarity between *Pr*_*MLCCOSMS*(32,4)_ and *Pr*_*TCP_trans*(4)_ was notable. Thus, MLCCOSMS has a good performance in countering inter-link correlation test.

## Discussion

MLCCOSMS inherits the multi-link covert modulation scheme of *Cloak* and embeds OSM in APL packet payload. Therefore, MLCCOSMS has the same performance of network overloads as that of *Cloak*. Theoretically, for larger *R* and *L*, the possibility of network overload becomes larger. This is because overfull concurrent APL packet flows and excessively long APL packet flows may cause network congestion. Besides, it is necessary to set APL packet sending rhythm of CS (denoted as *r*) more subtly to achieve better concealment. Therefore, we choose the strategy like [9] and set the *L* and *r* according to normal TCP flows (in fact, as MLCCOSMS supports staggered demodulation and breakpoint retransmission of the APL packets, *R* has no impacts on APL packet flow).

Besides the *unranking* and *ranking* algorithms [9] that use *O*(*n*) arithmetic operations for the covert modulation and demodulation, MLCCOSMS requires additional computing resources to address the synchronization marks. Since the size of mark is fixed, the computation time of *g*(*key*_*i*_) is fixed too. Assuming that a covert message block contains *N*_*w*_ codewords, thus the CS has to generate at least 2*RN*_*w*_ OSMs, and the computational complexity of OSMS generation for a covert message block is *O*(*n*) as *R* is also fixed. Similarly, the computation time required by *H*(*x*) to embed the three OSMs into each APL packet has a fixed maximum, because the max size of TCP payload is specified. Thus, the computational complexity at CS is *O*(*n*).

The computational complexity of *Exit*(*D*,**M**,*mac*) increases linearly with the size of **M**, in this light, its computational complexity is *O*(*n*), The algorithm 2 is mainly composed of two *Exit*(*D*,**M**,*mac*) for distinguishing the types of marks, Therefore, they exhibit *O*(*n*^2^) computational complexity. After that, two nested *Exit*(*D*,**M**,*mac*) is set to find ${\bar{key}}_{rand\_w\_j}^{seq'}$ and ${\bar{key}}_{i+r}^{seq'}$ by searching the *N*_*w*_ sets of ordinal marks (from ${\bar{M}}_{1}^{w'}$ to ${\bar{M}}_{N_{w}}^{w'}$) and the ${\bar{M}}_{j}^{c'}$ respectively. Suppose the total number of the ordinal marks must be iterated through at the *i*-th round of mark extraction is *M*_*i*_, then *M*_*i*+1_ = *M*_*i*_-1. Therefore, the the two nested *Exit*(*D*,**M**,*mac*) exhibit *O*(*n*^2^) computational complexity of extracting all the ordinal marks for a covert message block. As a result, the computational complexity at CR is *O*(*n*^2^).

Thus, the challenges pertaining to the improvement of the capacity, camouflage capability, reliability and undetectability of the MLCCOSMS, are converted to those regarding the demand of the computing resources of the CS and CR to implement *g*(*x*), *H*(*x*), and *Exit*(*D*,**M**,*mac*). This aspect is a cost-effective balance for the MLCCOSMS since the channel transmission resource is more valuable than the customizable host computing resource, as the covert channel is a parasite of the overt channel, has a narrow bandwidth and is vulnerable. However, the OSMS general applies to the MLCC with a moderate APL packet group size, as in certain covert communication scenarios, e.g., when using passive network cover channels, excessive consumption of the sender’s computing resources may arise the suspicion of the monitor.

## Conclusions

The passive synchronization schemes of *Cloak* lead to periodic suspension during packet transmission, which worsens in the presence of malicious interferences. Moreover, the synchronization schemes are rigid, and they result in the unconventional correlation of the packet transmission behaviours between the links. To solve these problems, this paper proposes the MLCCOSMS approach. MLCCOSMS obviates the dependence on ACKs and relieves the strict requirements for the order in which the links perform the sending behaviours as well as the sending order of the APL packets. Compared with those of *Cloak*, the throughput and reliability of the MLCCOSMS are higher. In addition, the MLCCOSMS considerably reduces the discontinuities of the APL packet transmission and minimizes the inter-link correlation of sending the APL packets. Moreover, the steganography approach of OSMS in MLCCOSMS can be proved to be safe.

Nevertheless, the MLCCOSMS needs to be improved. Since the increase in the covert codewords considerably increases the computational burden of the OSMS generation and covert modulation and demodulation, it is desirable to design a faster mark generation, organization and retrieval algorithm. Furthermore, it is still risky to run a covert channel between two fixed hosts for a long time. Therefore, deploying the MLCCOSMS on a distributed system based on cloud terminals can improve its concealment, undetectability and anti-traceback performances significantly.

## Supporting information

S1 Table Data of Fig 2. (XLSX)

S2 Table Data of Fig 3. (XLSX)

S3 Table Data of Fig 4. (XLSX)

S4 Table Data of Fig 6. (XLSX)

S5 Table Data of Fig 7. (XLSX)

S6 Table Data of Fig 8. (XLSX)

S7 Table Data of Fig 9. (XLSX)

S1 Appendix (DOCX)

## References

[1] Zkik K, Orhanou G, Hajji S E. Secure Mobile Multi Cloud Architecture for Authentication and Data Storage. International Journal of Cloud Applications and Computing. 2017;7(2):62–76.

[2] Zhang Q, Zhang X, Xue Y, Hu J. A stealthy covert storage channel for asymmetric surveillance VoLTE endpoints. Future Generation Computer Systems. 2019;102:472–480.

[3] Wendzel S, Zander S, Fechner B, Herdin C. Pattern-based survey and categorization of network covert channel techniques. Acm Computing Surveys. 2015;47(3):1–26.

[4] Xie H, Zhao J. A lightweight identity authentication method by exploiting network covert channel. Peer-to-Peer Networking and Applications. 2015;8(6):1038–1047.

[5] Zhang X, Chen L, Li QX, Zheng YZ, Tan J. Building covert timing channels by packet rearrangement over mobile networks. Information Sciences. 2018;445–446:66–78.

[6] Khan H, Javed Y, Mirza F, Khayam SA. Embedding a covert channel in active network connections. in Proc. GLOBECOM. 2009:1–6. doi: 10.1109/GLOCOM.2009.5425348

[7] Luo X, Chan EWW, Zhou P, Chang RKC. Robust network covert communications based on TCP and enumerative Combinatorics. IEEE Trans. Dependable and Secure Computing. 2012;9(6):890–902.

[8] Luo X, E. Chan EWW, Chang RKC. Cloak: a ten-fold way for reliable covert communications. in ESORICS, Lecture Notes in Computer Science. 2007:283–298.

[9] Luo X, Zhou P, Chan EWW, Chang RKC, Lee W. A combinatorial approach to network covert communications with applications in web leaks. in Proc. DSN. 2011:27–30. doi: 10.1109/DSN.2011.5958260

[10] Shi J. Steganalysis and detection of network covert channel Cloak. M.S. thesis, Dept. Automation, Nanjing university of science and technology. 2013.

[11] Wang H, Liu G, Shi J, Dai YW. A detection method for cloak covert channel based on burst size distribution. in Proc. CIHW. 2013;200–207.

[12] Watson D, Smart M, Malan G, Jahanian F. Protocol scrubbing: network security through transparent flow modification. IEEE/ACM Trans. Networking. 2004;12(2):261–273.

[13] Handley M, Kreibich C, Paxson V. Network intrusion detection: evasion, traffic normalization, and end-to-end protocol semantics. in Proc. USENIX Security Symp. 2001;10(9).

[14] Wang C, Yuan Y, Huang L. Base communication model of IP covert timing channels. Frontiers of Computer Science. 2016;10(6):1130–1141.

[15] Xie J, Chen Y, Wang L, Wang Z. A network covert timing channel detection method based on threshold secret sharing. Trans. Emerging Telecommunications Technologies. 2019;31(2).

[16] Giles J, Hajek B. An information-theoretic and game-theoretic study of timing channels. IEEE Trans. Information Theory. 2006;48(9):2455–2477.

[17] Qian YW, Sun T, Li J, Fan C, Song HJ. Design and analysis of the covert channel implemented by behaviors of network users. Security and Communication Networks. 2016;9(14):2359–2370.

[18] Archibald R, Ghosal D. A Covert Timing Channel Based on Fountain Codes. in TRUSTCOM. 2012;970–977.

[19] Iglesias F, Annessi R, Zseby T. DAT detectors: uncovering TCP/IP covert channels by descriptive analytics. Security & Communication Networks. 2016;9(15): 3011–3029.

[20] Cabuk S, Brodley CE, Shields C. IP covert timing channels:design and detection. in Proc. ACMCCS. 2004;178–187.

[21] Gianvecchio S, Wang H. An entropy-based approach to detecting covert timing channels. in IEEE trans. Dependable and Secure Computing. 2011;8(6):785–797.

[22] Zander S, Armitage G, Branch P. Stealthier inter-packet timing covert channels. in Proc. NETWORKING, Springer-Verlag. 2011;458–470.

[23] Shrestha PL, Hempel M, Rezaei F. Leveraging statistical feature points for generalized detection of covert timing channels. in Proc. MILCOM. 2014;7–11.

[24] Darwish O, Fuqaha A, Brahim GB, Athanasios IJ. Using hierarchical statistical analysis and deep neural networks to detect covert timing channels. APPLIED SOFT COMPUTING. 2019;82.

[25] Wang H, Liu GJ, Zhai JT, Dai YW. Detection and parameter estimation for jitterbug covert channel based on coefficient of variation. Ksii Trans. Internet & Information Systems. 2016;10(4):1927–1943.

[26] El-Atawy A, Duan Q, Al-Shaer E. A novel class of robust covert channels using out-of-order packets. IEEE trans. Dependable & Secure Computing. 2017;14(2):116–129.

[27] Zhang XS, Zhu LH, Wang XM, Zhang CY, Zhu HF, Tan Y. A packet reordering covert channel over VoLTE voice and video traffics. Journal of Network and Computer Applications. 2019;126:29–38.

[28] Zhang L, Huang T, Rasheed W, Hu X, Zhao C. An enlarging-the-capacity packet sorting covert channel. IEEE ACCESS. 2019;7:145634–145640.

[29] Swinnen A, Strackx R, Philippaerts P, Piessens F. ProtoLeaks: a reliable and protocol-independent network covert channel. in ICISS, Lecture Notes in Computer Science, Springer. 2012;119–133.

[30] Liu Y, Ghosal D, Armknecht F. Robust and undetectable steganographic timing channels for i.i.d. traffic. in Proc. IH, Springer-VerlagBerlin. 2010;193–207.

[31] Fall KR. TCP data flow and window management. in TCP/IP Illustrated Volume1 The Protocols, 2nd ed. Pearson Education. 2011;696–699.

