# Supplementary material for: Ordinal synchronization mark sequence and its steganography for a multi-link network covert channel
Source: PLoS One. 2021 Jun 4;16(6):e0252813. doi: 10.1371/journal.pone.0252813 (PMC8177511; doi:10.1371/journal.pone.0252813)
Supplement: S1 Appendix — (DOCX) [file pone.0252813.s008.docx]

**S1 Appendix**

Due to space limitations, the symbolic meanings and basic syntax used in the GNY logic are not presented in this paper, however, the readers can refer to literature [32] for the details. Herein, *Snd* and *Rev* correspond only to the CS and CR, respectively, and the APL packets sent by the CS are assumed to be completely received by the CR.

**1)Stage of generating $\overline{\mathbf{M}}$at the CS.**

The initialization assumption can be expressed as follows: , $g(x)\in Snd$, and . as the is randomly selected from . Thus, we obtain $\overline{key}_{1}^{seq}\in Snd$ and $Snd|\equiv\#(\overline{key}_{1}^{seq})$ by applying rules P4 and F10, among others to obtain $\overline{key}_{i}^{seq}\in Snd$ and $Snd|\equiv\#(\overline{key}_{i}^{seq})$.

**2)Stage of copying $\overline{\mathbf{M}}$ from the CS to the CR.**

Since the CS shares with CR before MLCCOSMS is established, and is the unique type mark of the negotiation, randomly selected from before the transmission process of the entire secret message block, it can be considered that and . According to the initial information shared between the CS and CR, we assume that the following logics hold at the beginning of this stage:

, , , , $Snd|\equiv\Phi(m)$,

, , , , $Rev|\equiv\Phi(m)$,

.

Proving that MLCCOSMS satisfies S2 and S3 is equivalent to proving the following four GNY conclusions:

G1:, , .

G2:, ,.

G3:, , .

G4:, .

--For G1, can be determined using $Data\in Snd$by applying rule P2, and can be determined by applying rule F1. Subsequently, we can obtain by applying rule F10. and can be proved in a similar manner.

--For G2, we can obtain and $Data\in Rev$ from the initial assumption by applying rule P1 and subsequently obtain by applying P2. Thus, it can be proved that , , and in the same manner as the proof for G1.

--For G3, by applying rule F1 on the initial assumption, it can be noted that , , , , and . Since has been proved in G2, we can deduce that , based on rule I3. Similarly, and can be reasoned.

--For G4, applying rule R1 yields, and can be proved by applying rule R5, as is already known. Similarly, we can prove that .

As $H(x)$ is a one-way hash function, the attackers cannot obtain the result of and without , therefore, even if $Data^{neg}$ is tampered or forged by the attackers, it cannot pass the verification of function $Exit(D,\mathbf{M},mac)$, thereby satisfying S4. *r* is used only to induce confusion to further increase the difficulty of attack.

**3)Stage of covert transmission.**

After stage 2), the CS shares all the marks and channel parameters with the CR, and a mark is not used once it has been used. Based on these aspects, the initial assumptions can be formulated as follows:

, , $\overline{key}_{rand\_w\_j}^{seq}\in Snd$, $Snd|\equiv\#(\overline{key}_{rand\_w\_j}^{seq})$, $\overline{key}_{i\text{+}r}^{seq}\in Snd$, $Snd|\equiv\#(\overline{key}_{i+r}^{seq})$, ,$Snd|\equiv\Phi(\overline{key}_{i}^{seq})$, , $Snd|\equiv\Phi(\overline{key}_{rand\_w\_j}^{seq})$, , , $\overline{key}_{rand\_w\_j}^{seq}\in Rev$, $Rev|\equiv\#(\overline{key}_{rand\_w}^{seq})$, $\overline{key}_{i\text{+}r}^{seq}\in Rev$, $Rev|\equiv\#(\overline{key}_{i+r}^{seq})$, , , $Rev|\equiv\Phi(\overline{key}_{rand\_w\_j}^{seq})$, $Rev|\equiv\Phi(\overline{key}_{i}^{seq})$, .

Similar to that in the previous stage, $H(x)$ and $Exit(D,\mathbf{M},mac)$ ensure that S4 is satisfied for every APL packet. Satisfying S2 and S3 is equivalent to proving the following four GNY statements:

G5:, $Snd|\equiv\#(H(Data||\overline{key}_{rand\_w\_j}^{seq}))$, $Snd|\equiv\#(H(Data||\overline{key}_{i+r}^{seq}))$.

G6:, $Rev|\equiv\#(H(Data||\overline{key}_{rand\_w\_j}^{seq}))$, $Rev|\equiv\#(H(Data||\overline{key}_{i\text{+}r}^{seq}))$.

G7: , $Rev|\equiv Snd|\sim H(Data||\overline{key}_{rand\_w\_j}^{seq})$, $Rev|\equiv Snd|\sim H(Data||\overline{key}_{i\text{+}r}^{seq})$.

G8:, $Rev|\equiv\Phi(H(Data||\overline{key}_{rand\_w\_j}^{seq}))$, $Rev|\equiv\Phi(H(Data||\overline{key}_{i}^{seq}))$.

--For G5, applying rule P2 on $Data\in Snd$ and yields . By applying rule F1, we obtain . Subsequently, we obtain by applying

rule F10. Similarly, $Snd|\equiv\text{\#(}H(Data||\overline{key}_{rand\_w\_j}^{seq}))$ and $Snd|\equiv\text{\#(}H(Data||\overline{key}_{i+r}^{seq}))$ can be proved.

--For G6, it can be inferred that and $Data\in Rev$, considering the initial assumption and rule P1. Subsequently, we can determine that by applying rule P2, and G6 can be proved in a similar manner as that of G5.

--For G7, applying rule F1 on the initial assumption yields , , and . As can be obtained by referring to G6, it can be concluded that by applying I3, and it can be proved that $Rev|\equiv Snd|\sim H(Data||\overline{key}_{rand\_w\_j}^{seq})$ and $Rev|\equiv Snd|\sim H(Data||\overline{key}_{i+r}^{seq})$.

--For G8, applying rule R1 yields . As is implied by G6, can be obtained through rule R5. Similarly, we can prove that $Rev|\equiv\Phi(H(Data||\overline{key}_{rand\_w\_j}^{seq}))$ and $Rev|\equiv\Phi(H(Data||\overline{key}_{i+r}^{seq}))$.

**Reference**

1. Li G, Needham R, Yahalom R. Reasoning about belief in cryptographic protocols. in IEEE Symposium on Security & Privacy. 1990;234-248.
